# Wetlands and Malaria in the Amazon: Guidelines for the Use of Synthetic Aperture Radar Remote-Sensing

**DOI:** 10.3390/ijerph15030468

**Published:** 2018-03-07

**Authors:** Thibault Catry, Zhichao Li, Emmanuel Roux, Vincent Herbreteau, Helen Gurgel, Morgan Mangeas, Frédérique Seyler, Nadine Dessay

**Affiliations:** 1 ESPACE-DEV, UMR 228 IRD/UM/UR/UG/UA, Institut de Recherche pour le Développement (IRD), 34093 Montpellier, France; emmanuel.roux@ird.fr (E.R.); vincent.herbreteau@ird.fr (V.H.); frederique.seyler@ird.fr (F.S.); nadine.dessay@ird.fr (N.D.); 2 Ministry of Education Key Laboratory for Earth System Modeling, Department of Earth System Science, Tsinghua University, Beijing 100084, China; zhichaoli@mail.tsinghua.edu.cn; 3 ESPACE-DEV, UMR 228 IRD/UM/UR/UG/UA, Institut de Recherche pour le Développement (IRD), SEAS-OI, 97410 La Réunion, France; 4 Department of Geography (GEA), University of Brasília (UnB), Brasília 70910-900, Brazil; helengurgel@unb.br; 5 ESPACE-DEV, UMR 228 IRD/UM/UR/UG/UA, Institut de Recherche pour le Développement (IRD), 98848 Nouvelle-Calédonie, France; morgan.mangeas@ird.fr

**Keywords:** wetlands, mosquito breeding sites, malaria, Amazon, SAR, remote sensing

## Abstract

The prevention and control of mosquito-borne diseases, such as malaria, are important health issues in tropical areas. Malaria transmission is a multi-scale process strongly controlled by environmental factors, and the use of remote-sensing data is suitable for the characterization of its spatial and temporal dynamics. Synthetic aperture radar (SAR) is well-adapted to tropical areas, since it is capable of imaging independent of light and weather conditions. In this study, we highlight the contribution of SAR sensors in the assessment of the relationship between vectors, malaria and the environment in the Amazon region. More specifically, we focus on the SAR-based characterization of potential breeding sites of mosquito larvae, such as man-made water collections and natural wetlands, providing guidelines for the use of SAR capabilities and techniques in order to optimize vector control and malaria surveillance. In light of these guidelines, we propose a framework for the production of spatialized indicators and malaria risk maps based on the combination of SAR, entomological and epidemiological data to support malaria risk prevention and control actions in the field.

## 1. Background of the Study

According to the WHO (World Health Organization) [1,2], vector-borne diseases, including mosquito-borne diseases, account for 17% of infectious diseases and are responsible for more than a million casualties each year. It is estimated that 41% of the world’s population (approximately 2.3 billion people) live in areas at risk of malaria [3,4] in 97 countries. The distribution of mosquito-borne infections is determined by complex dynamics involving environmental [5,6], social and economic factors. There is considerable concern regarding the potential impact of global change on the dynamics and spread of these diseases [7,8,9]. Malaria is one of the most common vector-borne diseases in the world with an estimated 214 million new cases and 438,000 deaths in 2015 [2].

On the American continent, the primary vector of malaria is the mosquito *Anopheles darlingi* (Root, 1926), which has widespread distribution in South America, including the Amazon Region [10,11]. Malaria in humans is caused by five species of parasites belonging to the genus *Plasmodium*. Previous studies frequently describe *An. darlingi* as a species that has a degree of dependence on the presence of forested areas [11,12,13,14,15] and the species is strongly dependent on the presence of water for its survival and dissemination [16]. Hence, it is crucial to identify and characterize wetlands’ distribution and dynamics in order to assess the processes involved in the transmission of malaria pathogens.

The breeding sites of *An. darlingi* can be natural (mainly riverbanks and flooded areas) or anthropic collections of water (including irrigated fields). In Belize (Central America), *An. darlingi* larval presence was positively associated with shade and submersed vegetation [17]. The suitability of wetlands for the development of vector larvae depends on various factors: shade, waterflow (stagnant water or water with movement), associated vegetation, the physical-chemical and bio-chemical conditions of the water and the distance to human populations [14,18,19,20]. The larvae of *An. darlingi* are thought to require stable chemical and physical conditions in their breeding sites, which are generally not found in small water bodies. This species preferably breeds in large, deep and clear water collections, such as lakes, swamps or large rivers [21]. Singer and Castro [22] considered the forest margins to be the principal breeding sites for *An. darlingi* in the Amazon. Deforested areas provide favorable conditions for malaria vector breeding and feeding; and forest and secondary forest can represent resting sites for adult mosquitoes that return to the forest and secondary forest after feeding [11,18]. It has also been proposed that deforestation and environmental changes imposed by human presence could increase *An. darlingi* breeding and malaria transmission [16].

## 2. Purpose of the Study

The control and prevention of malaria requires a better understanding and quantification of (i) the relative role of environmental factors (including land cover/land use, landscape features, meteorological and climatic factors) in the epidemic’s spread; (ii) the relative role of social and economic factors in these processes; and (iii) the vulnerabilities of populations to mosquito-borne diseases. It appears essential to understand the factors that cause increased vector densities and, hence, potential inequalities in the exposure to the transmission of pathogens. This can help to prevent the emergence and resurgence of more diseases, as well as to serve as a basis for effective control.

Remote-sensing techniques have been applied to epidemiology for decades [23]. In the Amazon region [24,25,26,27,28,29], remote-sensing techniques have been used for identifying mosquito habitats, investigating malaria epidemiology or assisting in malaria control (from larval habitat mapping at a local scale using aerial surveys to mapping the risk of transmission at a regional/continental scale using satellite data [30]). In 2000, Beck et al. underlined that the methodologies only became robust in the past 15 years with the development of many sensors (in terms of spatial and time resolution) and the growing use of field work associated with remotely sensed data. However, based on 438 research papers published in Landscape Ecology between 2004 and 2008, Newton et al. [31] emphasize that only 36% of the studies explicitly mention remote-sensing, only 5% are carried out at various scales, 3% use remote-sensing to develop new approaches of landscape characterization, 2% exploit multi-source data, and only 0.5% use very high resolution and/or SAR (Synthetic Aperture RADAR) data. Together with technical considerations, the extreme cost of acquiring such datasets at that time is another explanation for the underuse of SAR.

In this study, we choose to focus on the use of SAR remote-sensing for the characterization of wetlands and water collections that are the potential breeding sites of *An. darlingi*, in the Amazon region. Malaria primarily affects tropical and subtropical regions, where persistent cloud cover prevents the systematic use of optical remote-sensing for environmental studies. Unlike optical imagery, SAR sensors are independent of solar radiation and thus can capture images at night and through complete cloud cover [32]. Another advantage of SAR is the significant interaction of microwaves with water [33]. However, SAR-based studies in the literature are scarce and primarily focus on Africa [34,35]. Although limited in number, these studies clearly underline the interest in SAR for such diseases as malaria. Discussions with entomologists and epidemiologists led to the idea that the identification of breeding sites of the vectors requires the ability to detect and characterize various types of wetlands, including water bodies under tropical forest canopy and moist or temporarily flooded soils. This is clearly a challenge that can only be achieved using SAR data. For these reasons, this work proposes addressing the problem of the environmental characterization of *Anopheles* breeding sites through a review of SAR remote-sensing capabilities with a focus on the Amazon region.

In regard to health topics, remotely-sensed data can be under-exploited or used inadequately [36]. We often lack information regarding how to make remote-sensing data usable by bio-scientists or non-specialized users, and available remotely sensed information usually does not match epidemiological questions, nor is it adapted to the needs of public health actors. We intend to provide guidelines for using SAR remote-sensing regarding malaria vector control. More specifically, we intend to exemplify how the diversity (in terms of polarization, wavelength, time and spatial resolution) of currently available and future SAR sensors can be well-suited for (i) studying *Anopheles* breeding sites, such as natural wetlands and anthropogenic collections of water; and (ii) helping in the establishment of malaria vector control strategies.

## 3. Study Area: The Amazon Region

**Figure 1:**
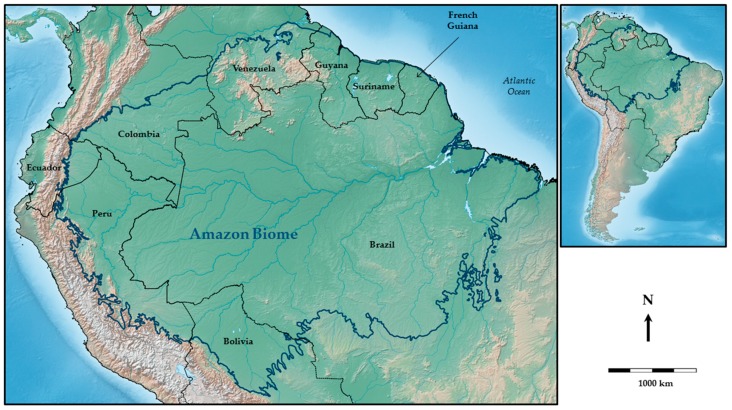
Geographical context of the study area in the Amazon region (the background is made with Natural Earth. Free vector and raster map data @ naturalearthdata.com, and the limits of the Amazon biome were obtained from the World Terrestrial Ecoregions map of the Nature Conservancy [38]).

This study’s area of interest is the Amazon region. This region is the largest tropical biome in the world (6.5 million km^2^), extending over nine countries (Bolivia, Brazil, Colombia, Ecuador, France/French Guiana, Guyana, Peru, Suriname, and Venezuela). The Amazon region today is inhabited by more than 30 million people, with a strongly heterogeneous population distribution throughout the territory [37]. It is the area most affected by *Anopheles*-borne diseases (Figure 1) in the American continent.

The Amazon Basin has the most extensive and diverse freshwater wetlands in the world. Amazonian wetlands range from small glacier-fed streams in the high Andes above 4000 m or more to the largest river in the world that is flanked by floodplain lakes, flooded forests and floating herbaceous communities.

**Figure 2:**
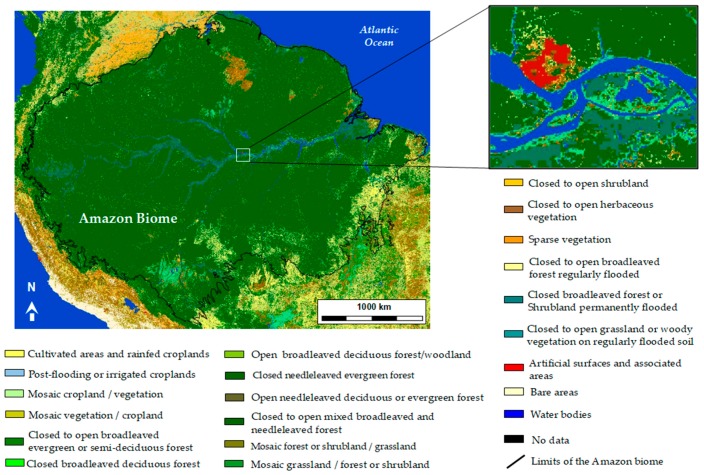
Land cover map (300-m resolution) of the Amazon biome extracted from the 2009 land cover world map produced in the framework of the ESA GlobCover project (credits: ESA/ESA GlobCover Project, led by MEDIAS France/Postel; see http://www.esa.int/spaceinimages/Images/2008/12/Envisat_global_land_cover_map for high-resolution access to the map and the legend).

Various worldwide medium- to low-resolution land cover maps have been produced in recent years that provide global knowledge regarding the distribution of wetlands, including those in the Amazon. For instance, in 2009, the European Space Agency (ESA), in collaboration with the University of Louvain (Belgium) and an international network of collaborators, produced a land cover world map using 19 months (from December 2004 to June 2006) of Envisat MERIS spectrometer images with a spatial resolution of 300 m. Figure 2 shows this land cover classification for the area of the Amazon biome. This land cover classification includes various classes of forest, vegetation and wetlands, which are of interest for our study regarding malaria and its vector environments/habitats.

Hess et al. [39] produced specific maps dedicated to wetlands inundation and vegetation for high water and low water seasons using JERS-1 SAR data, in which wetlands classes are different from the Globcover product, and include herbaceous vegetation, shrub, wood land and forest. These two examples illustrate the diversity existing in the classification and nomenclatures of wetlands, hence the importance to produce land cover maps and nomenclatures adapted to the needs of: (i) malaria epidemiology related to environmental factors and (ii) public health applications.

## 4. Role of Wetlands in Malaria Epidemiology

The influences of environmental risk factors on malaria vector development are well-established. Previous studies have shown evidence that local variation in hydrological processes, physiography, and land cover will influence the aquatic habitats for vector mosquitoes. In particular, the distribution of wetlands is critical regarding malaria epidemiology. Indeed, for a given malaria endemic area, anopheline mosquito production is spatially and temporally variable, and this variability is controlled by environmental conditions, such as the distribution of wetlands and conditions that can be detected with spaceborne sensors.

Stefani et al. [11] conducted a systematic review of the literature (17 papers), in which they studied the correlations between land cover classes and malaria risk in the Amazon. In particular, these researchers identified a class “water/wetlands” (consisting of deep water, shallow shady waters, fish ponds and wetlands in general) as a predominant risk factor for malaria transmission because it can form vector-breeding sites. However, this review showed that the relationship between wetlands and malaria risk is complex. This class is positively correlated with malaria risk in eight of the papers they reviewed, negatively correlated in two papers and of unknown correlation in seven papers. Water type can correspond to very different habitats, which may not be discriminated when using Earth observation data. This result led the authors to propose a model that demonstrated that landscape features play a key role in malaria risk construction by generating more or less favorable conditions for the encounter between mosquitoes and human beings.

The study by Stefani et al. [11] illustrates that in order to study the links between wetlands and malaria, the use of earth observation data has to be oriented toward the study of the space and time dynamics of wetlands in correlation with the dynamics of the vectors. The characterization of wetlands using remote-sensing (and SAR in particular) requires a focus on the dynamics of the various typologies of wetlands at various scales.

This characterization is a very complex process given: (i) the complexity of wetlands in terms of size, nature and geometry; (ii) the variations in space and time of wetland dynamics; and (iii) the diversity of SAR sensors, properties and techniques. In the next section of our study, we will focus on three types of wetlands: (i) free water (primarily rivers and lakes); (ii) water under mid-sized herbaceous-like vegetation (swamps, ponds, riverbanks, etc., including shrub vegetation); and (iii) water under forest canopy. We propose to provide guidelines for the use of SAR remote-sensing for wetland studies and review the advantages and limitations/uncertainties of currently available sensors. Next, we will proceed to review these sensors in regard to malaria surveillance and vector control.

## 5. Remote-Sensing for the Classification of Wetlands in the Amazon

Studying the links between *Anopheles* breeding sites (wetlands) and malaria pathogen transmission requires the correct identification and classification of the main breeding sites and thus to: (i) adapt the use of Earth observation data to monitor their dynamics in space and over time and (ii) consider wetlands’ characterization from the perspective of malaria processes.

Based on field investigations, Sanchez-Ribas et al. [20] proposed a “larval habitats” classification with seven categories based on seasonality, sun exposure, the presence of vegetation, and the susceptibility to water level fluctuations in the Brazilian Amazon. However, this classification was carried out at a local scale.

Classifying the entire Amazon region’s wetlands demands the use of specific tools, such as remote-sensing data. Sinka et al. [12] listed the main characteristics of malaria vectors’ larval sites in the Americas from a literature review of various data including Earth observation data. They classified Anopheles breeding sites according to their size, origin (natural or man-made water collections) and nature (e.g., lagoons, lakes, marshes, fish ponds irrigation channels, pools, wells, and borrow pits). They identified various key parameters describing each site such as light intensity, salinity, turbidity, water movement and vegetation (Table 1). Among the 41 species of Anopheles listed by Sinka et al. [12], only nine are present in the Americas. Among these nine species secondary malaria vectors in Amazonian countries include Anopheles marajoara (Galvão and Damasceno, 1942), braziliensis (Chagas, 1907), oswaldoi (Peryassu, 1922), nuneztovari (Galbadón, 1940) or trianulatus (Neiva and Pinto, 1922) [10,11].

### 5.1. SAR Remote-Sensing for the Characterization of Wetlands

The general awakening regarding the importance of studying, monitoring and conserving wetlands occurred in the 1970s and 1980s with the development of national programs, such as the National Wetland Inventory in the USA [40], the Canadian Wetland Inventory in Canada [41,42] and the signature of the international Ramsar Convention in 1971. These programs strongly benefited from the improvement of satellite remote-sensing (both optical and RADAR) techniques since the 1990s.

#### 5.1.1. Synthetic Aperture Radar: Theoretical Considerations

**Figure 3:**
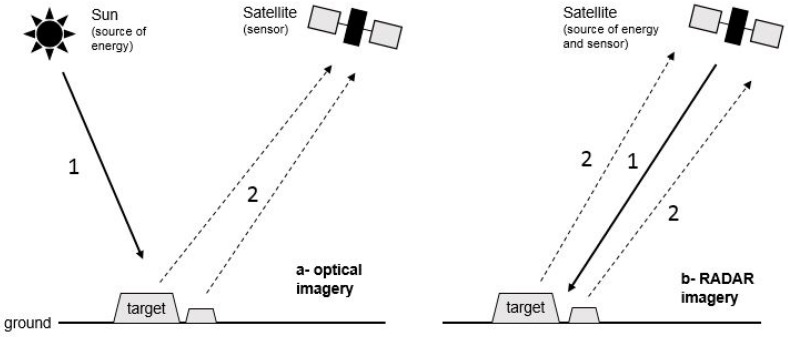
Principles of passive (optical) and active (SAR) satellite sensors. 1 = transmitted wave, 2 = received waves.

A synthetic aperture radar, or SAR, is a side-looking (airborne or spaceborne) active sensor that utilizes the flight path of the platform to simulate an extremely large antenna or aperture electronically, generating high-resolution remote-sensing imagery. Unlike optical sensors, SAR sensors generate their own source of energy, and the sensor receives the energy that is backscattered from the target surface (Figure 3).

Many SAR sensors are designed to transmit microwave radiation that is either horizontally polarized (H) or vertically polarized (V). A transmitted wave of either polarization can generate a backscattered wave with a variety of polarizations (vertical, horizontal, circular...). With SARs transmitting and receiving linear polarizations, there can be four combinations of transmit and receive polarizations:

- HH—for horizontal transmit and horizontal receive,
- VV—for vertical transmit and vertical receive,
- HV—for horizontal transmit and vertical receive, and
- VH—for vertical transmit and horizontal receive.

The first two polarization combinations are referred to as “like-polarized” and the last two combinations are referred to as “cross-polarized”.

Many SAR sensors currently are in operation (e.g., Sentinel 1A and 1B, Terrasar-X, Cosmo-Skymed, ALOS-2, and Radarsat-2) or are scheduled for launch in the next few years (Table 2). This diversity in sensors offers a wide range of possibilities in terms of spatial resolution, wavelengths, revisit periods and polarizations. In the past, accessing up-to-date SAR imagery could be costly, but it is now possible for all users to access Sentinel 1A and 1B SAR data for free. Much archived data (ERS 1 and 2, ALOS-1, etc.) are also available for free. This policy strongly encourages the use of SAR products for various applications, including public health.

Optical remote-sensing presents various limitations when focusing on wetlands [40,43,44,45] such as: (i) the fact that most wetlands are located in tropical or sub-tropical areas where cloud cover is frequent; (ii) possible confusion with other types of land cover; (iii) the strong difficulty in discriminating the various types of wetlands (e.g., vegetated, non-vegetated, level of water or moisture); and (iv) the fact that optical imaging cannot identify water bodies under vegetative cover and small streams.

Compared with optical remote-sensing, SAR has many advantages: (i) the possibility to acquire scenes day and night under any atmospheric conditions since the amplitude of the SAR signal is not strongly disturbed by cloud cover (L-band SAR are insensitive to atmospheric conditions while X-band SAR can experiment severe attenuation); and (ii) SAR sensors are well-adapted to the study of wetlands since the intensity of the backscattered signal strongly depends on the di-electric constant of the target (tropical wetlands and liquid water being a perfect targets).

Theoretically, SAR data have a strong potential for the study of wetlands, since the higher wavelengths of microwaves can penetrate vegetation cover and are sensitive to both soil moisture and the presence of open water under a canopy. The backscattered signal received by the SAR sensor from a wetland depends on: (i) the wavelength, polarization and incidence angle of the transmitted wave; (ii) soil roughness; (iii) vegetation biomass; (iv) dielectric properties of the ground and vegetation; and (v) the presence or absence of open water (inundation).

**Figure 4:**
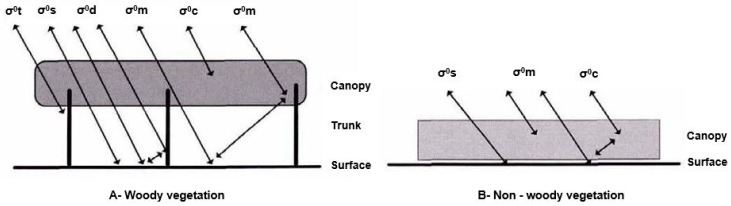
Sources of backscatter for vegetated wetlands in the case of (**A**) woody vegetation and (**B**) non-woody vegetation. $\sigma^{0}c$ = canopy backscatter, $\sigma^{0}d$ = soil-trunk backscatter, $\sigma^{0}m$ = soil-canopy backscatter, $\sigma^{0}s$ = soil backscatter, and $\sigma^{0}t$ = trunk backscatter (modified from [46]).

The choice of SAR sensor and polarization properties are of prime importance in order to be able to distinguish wetlands from the rest of the land cover types and to discriminate the various types of wetlands within the same ecosystem. Figure 4 summarizes the possible interactions between microwaves and vegetated areas for various types of wetlands [46].

Wang et al. [47] and Dobson et al. [48] showed that the total backscatter coefficient $\sigma^{0}tw$ for woody vegetation can be expressed as (Figure 4):

(1) $$\sigma^{0}tw=\sigma^{0}c+\sigma^{0}m+\sigma^{0}t+\sigma^{0}s+\sigma^{0}d$$

where $\sigma^{0}c$ = canopy backscatter, $\sigma^{0}d$ = soil-trunk backscatter, $\sigma^{0}m$ = soil-canopy backscatter, $\sigma^{0}s$ = soil backscatter, $\sigma^{0}t$ = trunk backscatter.

For non-woody (herbaceous) vegetation, Equation (1) becomes (Figure 4):

(2) $$\sigma^{0}th=\sigma^{0}c+\sigma^{0}m+\sigma^{0}s$$

The terms of these equations not only depend upon the type of vegetation covering the wetland but also upon the wavelength and polarization of the transmitted waves, the di-electric constant of the vegetation, the soil and water properties and the level of humidity of the wetland. Generally, the presence of water under a canopy tends to: (i) increase the total backscatter for a wetland covered by woody vegetation due to a double-bounce effect on vertical, vegetal structures; and (ii) decrease the total backscatter for a wetland covered in herbaceous vegetation.

#### 5.1.2. SAR and Wetland Typologies

Many reviews of SAR applied to the identification and characterization of wetlands have been conducted over the last several decades [49,50,51,52,53]. There are many types of wetlands [53], but they all have three primary characteristics in common: (i) the presence of water at the surface; (ii) very wet, shallow ground layers; and (iii) vegetation adapted to these types of conditions [54]. In our study, the primary concern is the presence or absence of vegetation associated with the water collections. The current diversity of the spatial resolutions of the main satellite sensors (from a few with resolutions of tens of meters to 10 m for low- to medium-resolution sensors, and reaching 1 m for very high-resolution sensors, Table 2) allowed us to carry out precise studies of water collections involved in the *An. darlingi* life cycle, ranging from large, permanent water bodies such as lakes or swamps to small, intermittent, domestic water reservoirs.

X-band, C-band, and L-band SARs have variable wavelengths and offer the possibility to penetrate more or less deeply into the vegetation cover. Microwaves only interact with objects whose size is the same order of magnitude as their wavelength, and a vegetation cover will be “seen” differently by SAR sensors. While the X-band data reflect on the top of canopy, C-band reflects on the canopy and branches (penetration to a few meters within the canopy), and L-band penetrates through the entire canopy to reflect on trunks, the water surface or ground surface [50,55,56,57,58]. In single polarization, HH polarization is generally more efficient than VV polarization to characterize vegetated wetlands [59,60,61,62]. Hereafter some recommendations regarding the identification of various types of wetlands using SAR are presented, and summarized in Table 3.

##### Detection of Free Water by SAR Sensors

Smooth water surfaces usually provide a specular reflection of microwave radiation, and, hence, very little energy is scattered back. In contrast, land surfaces scatter much more energy back to the radar due to, e.g., surface roughness and volume scattering. The difference in the energy received back leads to a high contrast between water and land. As a result of the radar’s unique response to water, water mapping using intensity thresholding methods on SAR images has been extensively used either with X-, C- or L-band SARs. However, X-band SAR is most suitable for mapping open water bodies and is better than options that use longer wavelengths, e.g., in the C- and L-band domains. With decreasing system wavelength, the sensitivity of a water surface to diffuse scattering increases [63]. Moreover, VV polarization is usually more sensitive to soil moisture and the presence of open water at the surface than HH or cross-polarization [56].

##### Detection of Water Covered in Herbaceous Vegetation

Several studies showed that C-band is more appropriate for the detection of moist or temporary inundated soil under agricultural cover or herbaceous vegetation [56,57,58], since the wavelength of C-band allows the penetration of herbaceous vegetation, while X-band SARs only detect the vegetation layer covering the water. Kasischke et al. [50] proposed a review in which they identified that like-polarized SARs are well-suited for the detection of flooded vegetation, with C-HH being preferred for herbaceous wetlands. Karszenbaum et al. [61] investigated the possibility of determining the hydrological conditions of wetlands in the Parana River Delta (Argentina) using multi-source and multi-temporal SAR data. They tested the influence of sensor features on the backscattered signal as a function of hydrological conditions, comparing ERS-2 C-VV and RADARSAT-1 C-HH data. The results showed that unlike RADARSAT-1 in HH polarization, ERS-2 in VV polarization is able to detect changes in hydrological conditions (variable levels of inundation) in swamp areas and areas covered by bulrush-type vegetation.

##### Detection of Water under Forest Canopy

In tropical regions, L-band sensors are more suitable than X-band and C-band sensors for the identification of wetlands covered by forest or dense herbaceous vegetation. Although X-band and C-band are more limited in such cases, C-band SAR can be useful when the canopy is sparse or during seasons when leaves are absent [50,55,56,57,58].

The combination of C-band and L-band SAR is an efficient tool for the detection of water under canopy [56]. Because L-band sensors are weakly sensitive to smooth surfaces, they generally allow the discrimination between inundated forest and non-inundated forest areas [49]. Townsend [57] added that the multi-temporal use of RADARSAT-like SAR data together with JERS-1 (L-band) and ERS-1 (C-band) data can be sensitive to the density of the forest covering wetlands and canopy height.

In Kasischke et al. [50], like-polarized SARs appear well-suited for the detection of flooded vegetation, with L-HH being preferred for wooded vegetation. In C-band, the HH-polarized RADARSAT-1 signal produces a backscatter signal strongly influenced by the presence of water under woody vegetation, while swamps always have a similar signal regardless of the hydrological conditions [50]. Hess et al. [49] showed that: (i) wetland detection under a canopy is facilitated by incidence angles inferior to 35° and (ii) a multi-incidence approach might help identify various vegetation structures within the same wetland system.

##### Discriminate Wetland Classes Using SAR

The combination of C-band and L-band SAR allows for the precise cartography of wetlands [60]. Crossed polarization (VH or HV) is usually more adapted for the distinction between wetlands with woody vegetation and wetlands with herbaceous vegetation due to the sensitivity of cross-polarization to the biomass [52]. Hess et al. [49] used the satellite SIR-C to clearly show that together with the combination of C and L frequencies, the combination of polarizations is an efficient tool for the identification of vegetation types of wetlands and for mapping wetlands [62].

Multi-polarized imagery improves the classification of wetlands compared with mono-polarized imagery. This enhancement is particularly true in areas where vertical vegetation, such as bulrush and herbs, are mixed with trees and shrubs with branches that are more randomly distributed [60]. Polarimetric SAR offers a wide range of polarimetric features through multi-frequency views, multi-polarizations, and a wide range of polarimetric descriptors correlated to the biophysical and geometrical characteristics of the various surface conditions defined by the backscatter mechanism of ground targets. Patel et al. [63], based on DLR-ESAR (Indian experimental mission) L- and P-band data, used polarimetric decomposition to understand the backscatter mechanisms associated with wetlands in those bands. The results showed that such decomposition can discriminate various systems composing the same wetland and allow the realization of wetlands’ classifications. Similarly, comparisons between bi-polarized C-band SAR products and fully polarimetric products from the airborne sensor CV-580 provide relevant information [64]: (i) the combination of HH polarization and the ratio HH/HV is the most efficient method to discriminate flooded vegetation from unflooded vegetation and open water; (ii) Freeman-Durden decomposition clearly identifies flooded vegetation caused by the double-bounce backscatter appearing brighter on the images; and (iii) Cloude-Pottier decomposition allows an initial classification of the vegetal species composing the wetland.

##### Monitor Flooding Conditions and Water Level Variations in Wetlands Using SAR

Schmitt et al. [65] used one of the parameters of the Cloude-Pottier decomposition (alpha angle, characterizing the predominant backscatter mechanisms) in a multi-temporal approach to change detection on RADARSAT-2 data. Long term monitoring of flooded vegetation was carried out and revealed that: (i) a single image at a single date is enough to identify wetlands; and (ii) using a temporal series of images and comparing alpha angle values shows that backscatter mechanisms can be directly correlated to floodings or drought events affecting a wetland.

While many studies address the use of SAR signal amplitude or polarimetry, SAR interferometry (InSAR) uses two or more SAR images to generate maps of surface deformation or digital elevation using differences in the phase of the waves returning to the satellite. InSAR is usually used to study the changes of solid surfaces, but it is also capable of detecting changes in water height in the presence of emergent vegetation [66,67]. InSAR can provide a high-resolution hydrological map of wetlands that cannot be obtained using ground methods [67], but this method is only efficient in an aquatic environment in which vegetation is emergent since the combination of horizontal water surfaces and vertical vegetation offers the optimum conditions for the double-bounce mechanism [67]. This method works for any SAR frequency provided that the interval between two successive images (temporal baseline) is low [67,68,69,70]. According to Hong et al. [69,70], interferograms calculated in cross polarizations (VH or HV) seem more adequate in order to characterize water level changes in wetlands. The coherence of interferograms is sensitive both to the type of vegetation of the wetland and the time interval between the two images. If this interval is inferior to 6 months, InSAR can detect water level changes for various types of wetlands, but better results are obtained with L-band sensors in HH polarization with a low temporal baseline [67,69]. For RADARSAT-2, Hong and Wdowinski [71] showed using the example of the Everglades wetlands (Florida, USA), that the HH polarization with a baseline inferior to 48 days (two revisit cycles) are the best conditions to apply efficiently InSAR to wetlands, the C-band being less sensitive to atmospheric effects than X-band and to ionospheric effects than L-Band.

Kim et al. [72] carried out an excellent study of water level variations in the wetlands of Louisiana (USA) that combined C-band (RADARSAT-1) and L-band (PALSAR) InSAR with altimetry (ENVISAT) in order to: (i) monitor high-resolution water level changes; (ii) detect main directions of water flux within the wetland; (iii) identify discontinuities in this water flux; and (iv) create high-resolution water flux models. Coherence analysis of InSAR pairs suggested that the HH polarization is preferred for this type of observation. Research by Lee and Pottier [73] using TOPEX/POSEIDON altimetry in Louisiana confirmed the results obtained by Kim et al. [72] with ENVISAT altimetry. TOPEX is capable of measuring accurate water level changes beneath a heavy vegetation canopy region (swamp forest). It is to expect that the new generations of satellite-borne altimeter/interferometer (as Siral in the Cryosat 2 mission, or the future SWOT mission) will be particularly adapted (fine resolution and swath mode compared to the spot return of the nadir radar altimeters).

##### Summary of Recommendations for the Use of SAR to Characterize Wetlands

Table 3 summarizes the guidelines regarding the use of SAR properties and techniques for the detection of free water, herbaceous-vegetated wetlands and wetlands under forest canopy.

**Figure 5:**
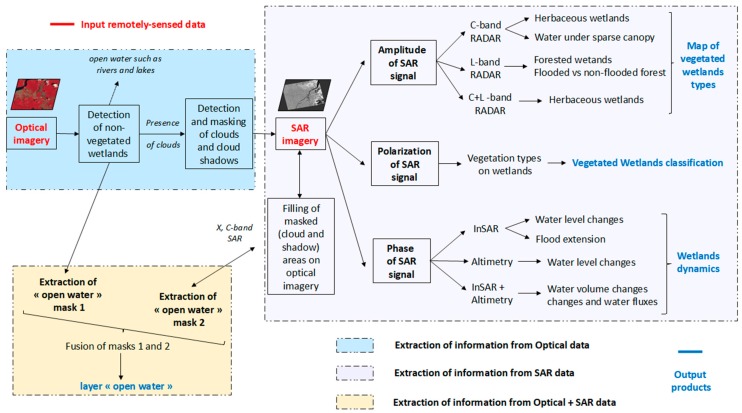
Proposed framework for the characterization and mapping of wetlands and water collections by combining optical and SAR remotely sensed data.

On the basis of these recommendations, Figure 5 proposes a framework based on SAR earth observation data for the production of wetland classification maps. In this framework, the layer containing free water information is extracted from the combination of optical imagery and X-band or C-band SAR. Thresholding over SAR amplitude can be used to discriminate and map wetland types by combining C- and L-band SARs. The polarization of SAR signals provides information regarding the vegetation type over the wetlands and helps refine the classification based on amplitude thresholding. Finally, water level fluctuations and changes in flooding conditions can be quantified using phase techniques such as InSAR or altimetry, thereby permitting the classification of wetlands based on seasonal dynamics.

**Figure 6:**
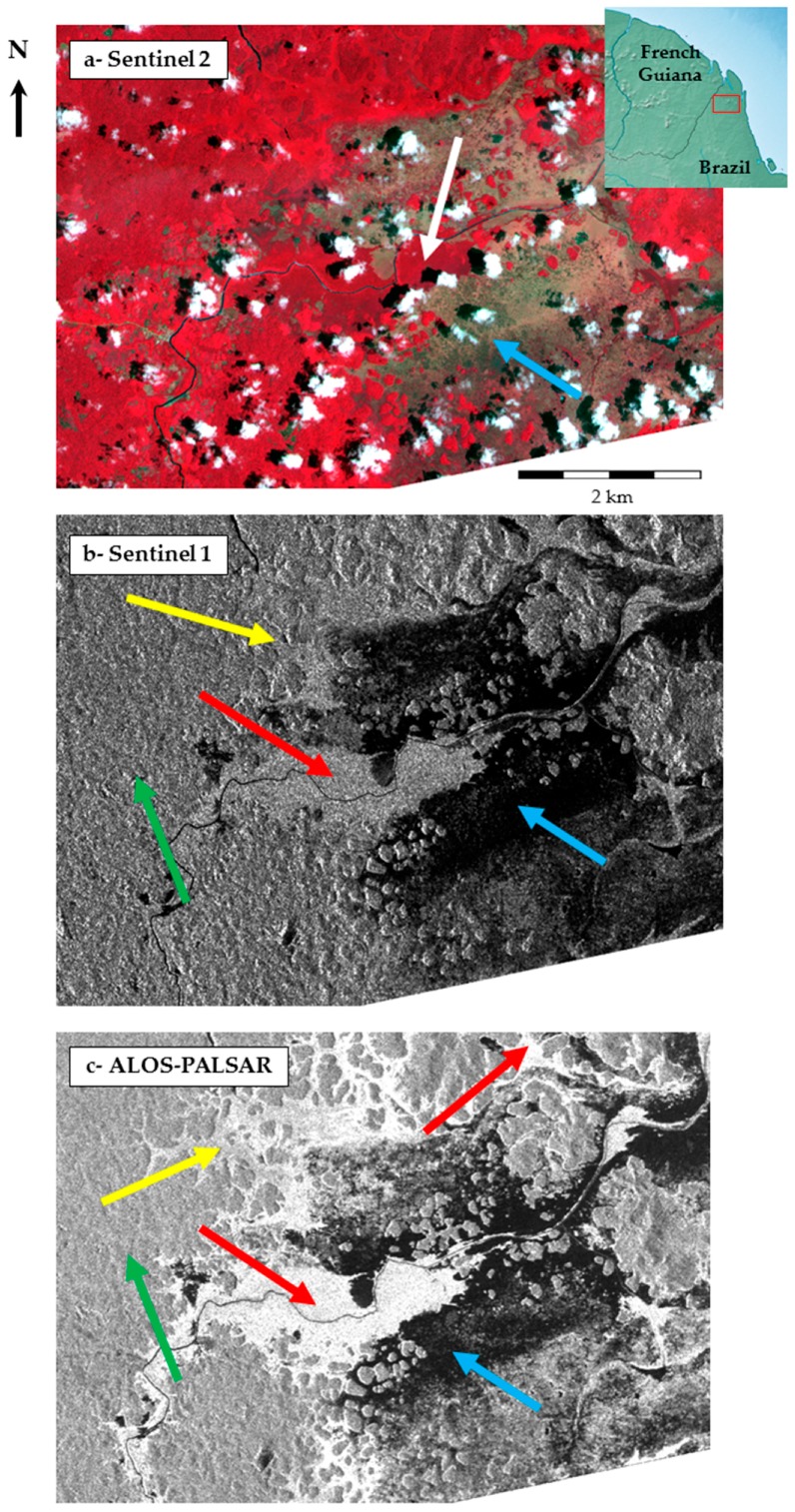
Comparison of reflectance and backscatter signal from Sentinel 2 optical image (**a**) dated September 2016, © European Space Agency—ESA, 2016, C-band SAR Sentinel 1 in VH polarization; (**b**) dated May 2016, © European Space Agency—ESA, 2016 and L-band SAR ALOS PALSAR in HH polarization; (**c**) dated May 2010, © JAXA, METI, 2010) on a wetland area in the Amazon, at the border between French Guiana and Brazil.

Figure 6 compares the signal (optical reflectance and SAR backscattering) of the same area covered by vegetated wetlands in the Amazon at the border between French Guiana and Brazil. Using Sentinel 2 data at 10 m resolution (a), the observed surface exhibits a typical signal of vegetation (white arrow), while bare soils are identified by a blue arrow. In contrast, when imaging the same area with SAR sensors, C-band Sentinel 1 at 10 m resolution in VH polarization (b) allows the discrimination between free water in black (specular reflection showed by a blue arrow, but forested wetlands (flooded forest, red arrows) are not strongly discriminated from non-flooded forest (green arrow) and herbaceous wetlands (yellow arrow). The use of ALOS-PALSAR L-band sensor (JAXA, Tokyo, Japan) at 12.5 m resolution in HH polarization (c) however is capable of evidencing free water (blue arrow), non-flooded forest (green arrow), herbaceous wetlands (yellow arrow) and more importantly, flooded forest (red arrow) with very elevated backscatter coefficient values (in white) due to the double-bounce mechanism (double backscatter resulting from the reflection on trunks and water surface located below the canopy, see Figure 4A).

### 5.2. SAR for Wetlzand Classification in the Amazon Region

From 1973 to 1985, the Brazilian government funded the project RADAMBRASIL (Radar da Amazônia do Brasil) dedicated to the mapping of natural resources based on airborne SAR data. Later, other studies used SAR data to produce wetland maps in the Amazon region using various sensors, such as SIR-C (NASA/JPL, Pasadena, CA, USA), JERS-1 (JAXA, Tokyo, Japan) ALOS-PALSAR (JAXA, Tokyo, Japan), RADARSAT-2 (CSA, Mac Donald Dettwiller Geospatial Services Inc., MDA GSI, Quebec, QC, Canada) or RADAR altimetry data from ENVISAT (ESA) or TOPEX-POSEIDON (NASA/CNES). Table 4 summarizes these studies, indicating the type of SAR technology used, the main results and the resulting products.

Table 4 shows that the diversity of SAR sensors (C-band, L-band, SAR properties (e.g., wavelength and polarization) and techniques (backscatter, texture, interferometry, altimetry) is exploited in order to characterize the complex ecosystems of Amazonian wetlands. Figure 6 is an example of the wetland mapping obtained using SAR in the Amazon basin [82] where wetlands are classified according to the vegetation type (herbaceous vegetation, shrub or forest) and the state of inundation in low-water and high-water seasons.

**Figure 7:**
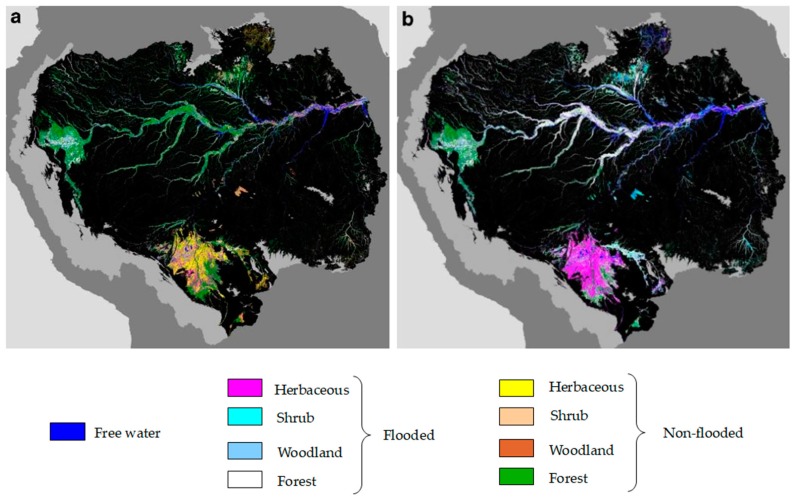
(**a**) Amazon wetland classes mapped during October–November 1995 (low water) and (**b**) May–June 1996 (high water). Black areas are non-wetland areas, and gray areas within the Amazon basin have elevations greater than 500 m (from [39]). The legend of this figure has been slightly modified to refer directly to the classes identified in Table 3.

In the frame of the Global Rain Forest Mapping project, wetland extent, vegetation cover, and inundation state were mapped for the first time at moderately high (100 m) resolution for the entire lowland Amazon basin, using mosaics of Japanese Earth Resources Satellite (JERS-1) imagery acquired during low- and high-water seasons in 1995–1996. Hess et al. [39] applied a rules set to the GRFM map to classify wetland areas into five land cover classes and two flooding classes using dual-season backscattering values. The mapped wetland area of 8.4 × 105 km^2^ is equivalent to 14% of the total basin area (5.83 × 106 km^2^) and 17% of the lowland basin (5.06 × 106 km^2^). During high-water season, open water surfaces accounted for 9% of the wetland area, woody vegetation 77%, and aquatic macrophytes 14% (Figure 7).

**Figure 8:**
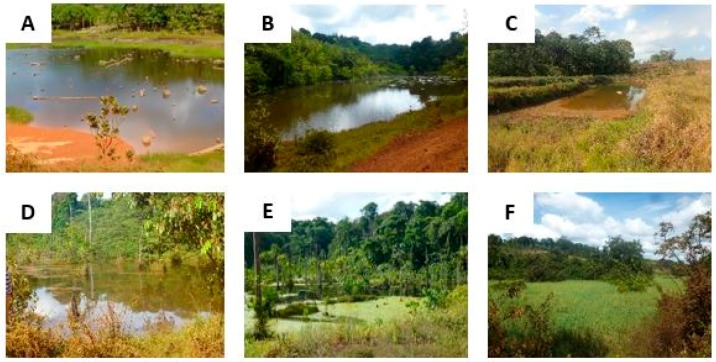
Various types of vegetated wetlands encountered in the Amazon region, more specifically in a rural forested area. (**A**,**B**) = poorly vegetated ponds; (**C**) = man-made water reservoir located at the fringe of the forest; and (**D**–**F**) = poorly to strongly vegetated swamps with few trees and dense herbaceous vegetation.

**Figure 9:**
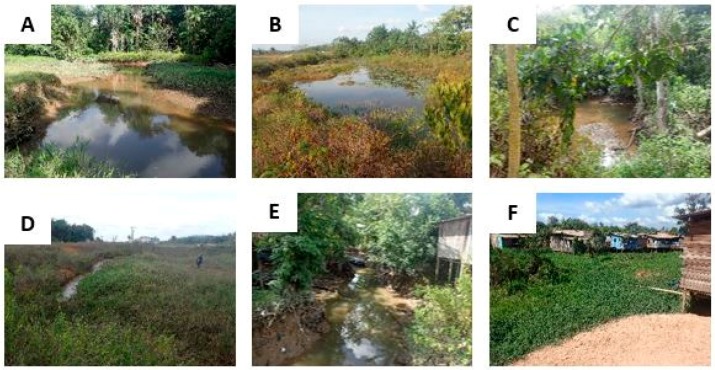
Various types of vegetated wetlands encountered in the Amazon region, more specifically in a peri-urban area. (**A**) = riverbed with vegetated edges; (**B**) = heterogeneous vegetation at the surface of a pond; (**C**) = roadside muddy ditch; (**D**) = roadside ditch composed of stagnant water; (**E**) = riverbed where freshwater and used water are mixed; and (**F**) = strongly vegetated swamps surrounded by wooden houses on stilts.

Typical Amazon wetlands are presented in Figure 8 and Figure 9. These photos illustrate ponds, swamps, ditches or water reservoirs more or less densely covered in vegetation in a rural to peri-urban environment at the cross-border area between French Guiana and Brazil. Referring to Table 1, the wetlands presented here are suitable for all the species of *Anopheles* in the table. However, while some wetlands are entirely covered in vegetation at their surface (Figure 8E,F and Figure 9F), some others only exhibit vegetation on their edges (Figure 8B and Figure 9B).

The distribution of *Anopheles* larvae in these wetlands is therefore likely to be different. Figure 9D,E show ditches were the water quality is low, hence being more suitable to *An. albitarsis*. Sun exposure is also very variable: apart from Figure 9C,E, most wetlands are strongly exposed to the sun, except on the edges where trees offer shade. This will also influence the distribution of larvae and the suitability of such wetlands to *Anopheles* larvae, most species (except *An. albitarsis*) being more adapted to low sun exposure (Table 1). However, for all these wetlands, water is stagnant and suitable to all the species of Table 1.

## 6. SAR Remote-Sensing of Wetlands and Malaria Control in the Amazon

### 6.1. Previous Studies

Few studies directly address the connection between environmental features and diseases using SAR data in the Amazon. The connection between the environment, optical remote-sensing and malaria, for example, is very well shown by Stefani et al. [11]. Hay [32] reviewed some applications of SAR remote-sensing to vector-borne diseases and public health. Ross et al. [34] and Kaya et al. [35] first underlined the potential of RADARSAT-1 and 2, respectively, in investigations related to the environment and vector-borne diseases. In his review of remote-sensing applications as tools for monitoring endemic diseases in Brazil, Correia [26] “found no reference to radar, although they are mentioned in some articles as a potential resource for detecting floodable areas, possible habitats for mosquito larvae”. Olson et al. [83] mapped the maximum extent of wetlands and water collections using JERS data in order to quantify the influence of precipitation, floods and landscape on the geographical distribution of malaria risk in the Amazon region in the framework of climate change. Li et al. [83,84] combined optical SPOT 5 and SAR PALSAR images on one hand and DEM with PALSAR data on the other hand at the border between French Guyana and Brazil to identify the environmental features involved in the dynamics of malaria in the area, such as land cover (urban areas, forested areas, wetlands and the interfaces between them), geomorphological features and soil typologies to access the breeding sites of malaria vectors and the water’s physical and chemical properties.

### 6.2. From Wetland Characterization to Malaria Exposure Risk in the Tropics

Quantifying the distribution of wetlands and the variations of wetland dynamics in space and time is the first step in the assessment of malaria risk associated with environmental factors.

(1) Producing multi-temporal maps of wetlands and water collections is of prime importance in order to identify potential suitable habitats for the vectors of malaria (the hazard component of malaria risk). The identification of various types of water collections combined with exhaustive field sampling allows us to discriminate the types of wetlands more adapted to specific species of *anopheles* vectors [12]. Updating those maps over time, especially given the changes occurring between dry season and rainy season in tropical regions (changes on vegetation cover or in water extent, leaving extensive moist soils and fragmented pools when the wetland dries up), is primordial in order to survey how the geographical distribution of larval reservoirs evolves over time. Once potential larval reservoirs have been identified, the estimation of habitat suitability for vectors can be carried out through species distribution models for example [85]. Together with wetland mapping and the evolution of wetlands in space and time, the definition of vector control strategies relies on the estimation of wetlands’ larval production. This step, based on field sampling, is primordial in order to correctly assess the role of wetlands in malaria risk. The seasonality of environmental factors estimated using remotely sensed data echoes in the predictions of malaria seasonality (the combination of disease risk in space and time).
(2) Water level changes within water collections can be associated with heavy rain fall and flooding typical of tropical regions such as the Amazon. The dynamics of water stage imply dynamics in vector populations [86]. While some species of *anopheles* are adapted to either still, stagnant or turbulent water collections, some others have a specific affinity for non-disturbed waters. When water level decreases, the length of water collections margins increases, by the effect of pools fragmentation, combining dense vegetation and moist soils and therefore creating suitable conditions for the development of larval reservoirs [12]. Discriminating between areas covered by open water and areas with high soil moisture is a strong concern for entomologists since both types or areas can be larval reservoirs and their discrimination is not easy using satellite imagery (either optical or SAR images).
(3) In the context of global change (either climate change of human-induced changes), land cover and wetland distributions are strongly modified, thereby causing changes in the geographical expansions of vector-borne diseases and exposing new populations to disease [87]. Efficient prevention and vector control depends on the availability of up-to-date data and land cover maps combined with malaria case databases (such as the SIVEP database in Brazil [88]) at the local and regional scales in order to support targeted interventions. In many countries, national malaria control programs lack detailed disease-risk maps to guide intervention and reinforce vector control. Such maps could: (i) optimize the use of larvicide in targeted areas where the presence of suitable candidate wetlands and population has been acknowledged; (ii) optimize the timing of insecticide distribution for impregnating bed nets; (iii) restrict the distribution of antimalarial drugs to periods of known disease risk; (iv) help defining local environmental engineering actions and (v) help defining more specific prevention messages.

**Figure 10:**
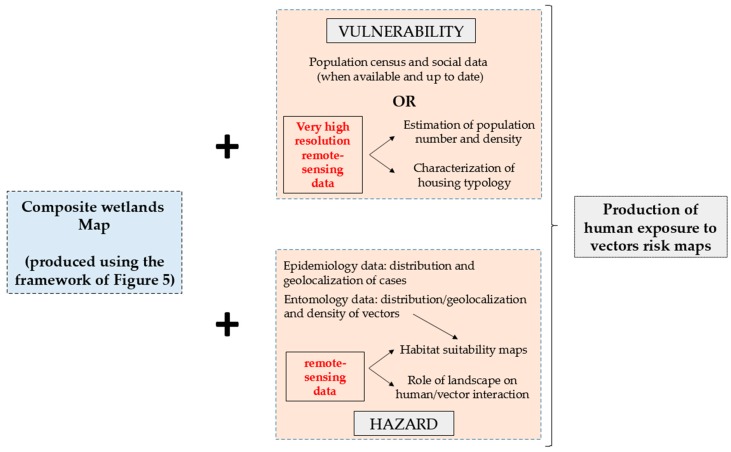
The contribution of remote-sensing and land cover mapping using optical and SAR data in the definitions of hazard and vulnerability for the production of human exposure to vector risk maps.

The production of malaria risk maps is not only based on vector ecology and environmental factors (hazard “box”, Figure 10) but also on factors involving human populations (“vulnerability box”, Figure 9). For instance, the distance between human settlements and water collections is one of the main parameters controlling the risk of exposure of the population to the vectors [89]. Figure 9 summarizes the contribution of remote-sensing in the characterization of hazards (related to the vectors) and vulnerability (related to the human population) on the basis of environmental variables extracted from land cover maps produced using optical and SAR data. For factors regarding the “hazard box”, remote-sensing can be exploited to realize habitat suitability maps [85] and the calculation of the landscape-based hazard index of human/vector interaction [15]. To overcome the lack of information regarding human populations (number of inhabitants, population density, etc.) in the “vulnerability box”, optical and SAR very high-resolution images could provide an estimate of population numbers and density from the characterization of housing typologies.

Combining these two “boxes” of hazard and vulnerability seen from the remote-sensing point of view, it is possible to consider a first approach of the human exposure to vector risk.

This remote-sensing-based approach echoes the review carried out by Cohen et al. [90] in which mathematical models are exploited in order to: (i) map and evaluate malaria metrics related to a variety of risk components relating to the malaria transmission cycle and (ii) assess the implications of these components in the decision-making process in the frame of health policies.

### 6.3. Improving the Characterization of Wetlands to Improve Vector Control

Despite discrepancies between published articles, it is evident throughout the recent literature that multi-dimensional SAR data sets are achieving an accepted role in operational situations that require information regarding wetland presence, spatial extent and characteristics in time. We strongly believe that with the development of increasingly numerous SAR sensors and the growing possibilities offered in terms of free access to the data (such as Sentinel 1), image processing freeware and user community growth and expertise, this technology has a strong future regarding the monitoring of vector-borne diseases and, more generally, public health.

From the perspective of remote-sensing, improving our knowledge of wetland dynamics in space and time will improve our capacity to build efficient vector control strategies. In this effort, future SAR sensors have a key role to play in the improvement of wetland mapping. The future of SAR satellites for forests and wetland studies lies in the use of P-band sensors, with wavelengths reaching 30 cm to 1 m that provide the possibility for electromagnetic waves to see through the entire canopy in order to count tree trunks and reach the ground below forests. Due for launch in 2020, the BIOMASS satellite will carry the first P-band SAR with the ability to: (i) deliver accurate maps of tropical, temperate and boreal forest biomass; and (ii) penetrate deep into the forest cover to detect water bodies under dense canopy. Hence, the BIOMASS satellite presents a great potential for the identification of mosquitoes’ breeding sites and applications to public health. The Surface Water & Ocean Topography (SWOT) mission is scheduled for launch in 2021. By combining SAR Altimetry and InSAR techniques, this new space mission is designed to make the first global survey of the Earth’s surface water, observe the fine details of the ocean’s surface topography, and measure how water bodies change over time. By measuring water storage changes in all wetlands, lakes, and reservoirs and making it possible to estimate discharge in rivers more accurately, SWOT will contribute to a fundamental understanding of the terrestrial branch of the global water cycle. SWOT will also map wetlands and non-channelized flow, and it appears to be a highly promising sensor for applications in public health related to vector-borne diseases.

### 6.4. Limitations of SAR Remote-Sensing for Public Health Topics Remain

Although remote-sensing is suitable for the study of environment-related vector-borne diseases, several limitations remain, and many investigations are still needed to fully understand the mechanisms involved in the relationship between environmental features and vector-borne diseases. Improving satellite sensors will not resolve all issues regarding the use of remote-sensing for public health. Here, “a better understanding of the techniques and methods needs a close collaboration between epidemiologists and geographers” [36].

#### 6.4.1. Multi-Scale Problem

Bio-ecological phenomena such as vector-borne diseases exhibit patterns at different scales [9]. The diversity of studies we reviewed show that some authors study vector-borne disease at a regional scale while others focus on a local scale. One of the key to study the relationship between environment and vector-borne diseases is to find out at what scale operate the processes involved, in order to determine the most suitable (or suitable combination of) earth observation data. Multi-scale studies usually are extremely difficult to carry-out. The diversity of scale echoes in the diversity of current remotely-sensed data, from low-resolution sensors (some hundreds of meters to kilometer resolution) operating at a regional scale (meteorological sensors from NOAA for example) to very high resolution sensors zooming (with a resolution around 1 m) at a very local scale (recent optical and SAR sensors developed in the last ten years, such as SPOT 6/7, TerraSAR-X…). Resolution diversity can also apply to time resolution and spectral resolution. Before addressing an environmental problem, one needs to make sure the resolutions (space, time and spectral) of the remotely-sensed data are suitable to the scale of the biological, physical or ecological process under study.

The scale (from local to regional, national or continental) and the spatial resolution of images (defining the size of the smallest possible feature that can be identified using satellite imagery), are important considerations in the representation of malaria risk and the definitions of vector control strategies [91].

#### 6.4.2. Lack of Ground Data

Models of malaria risk integrate entomology and epidemiology data to produce disease transmission hazard/risk maps. There is usually a considerable discrepancy between the amount of remote-sensing data available compared with the entomological and epidemiological data on the studied disease in the studied area. In some countries, medical systems can be severely inadequate, and cases of vector-borne diseases are not reported or spatialized. Moreover, the number of malaria cases is not sufficient to study transmission processes; knowledge of its incidence is required, but fine demographic data at spatial resolutions compatible with local studies of vector-borne diseases are scarce or non-existent. To successfully apply remote-sensing to the surveillance, prevention, and control of vector-borne diseases, we need well-designed surveillance systems to provide the “ground truth” data necessary to validate the models being developed. This is clearly a gap that needs to be filled in order to develop early warning systems dedicated to malaria.

#### 6.4.3. Public Health Actors’ Needs vs. Space Agencies’ Requirements

The fact that many sensors are being developed by space agencies for future research applications is a good point for the scientific community. It is even better news when such programs as Copernicus offer free access to high quality optical and SAR data, and when increasingly abundant archived data become free of charge (SPOT World Heritage program, for example). However, it is of prime importance that the technical capacities of future sensors match the needs of users (e.g., scientific community, local authorities, and governmental agencies) as closely as possible, especially in such domains as public health (i.e., the development of “thematic” sensors).

## 7. Conclusions

The utility of SAR remote sensing and other earth observation data for improving the understanding, prevention, and control of malaria has been abundantly demonstrated. Given the current activity of the SAR community and the need to overcome cloud cover issues in tropical regions where most vector-borne diseases occur, SAR is becoming an increasingly productive tool for the monitoring and surveillance of environmental processes, including those related to public health. Despite several remaining limitations, we showed that the diversity in the technical capacities of RADAR sensors makes this technology well-adapted for the study of wetlands and water collections. In this sense, we provided guidelines and recommendations for the use of remote-sensing techniques based on SAR imagery to study the relationship between remote-sensing, wetlands and mosquito-borne diseases, such as malaria.

For taking a step forward it is necessary to implement operational systems that facilitate real-time monitoring of human health. This step requires to bring together researchers from various fields of expertise in pluri-disciplinary approaches involving the remote sensing community, entomologists, epidemiologists, medical doctors, GIS specialists … in large scale projects. Some initiatives like that of the health observatory or “sentinel” sites at the cross-border between French Guiana and Brazil [92] represent a first effort towards the development of reinforced monitoring and surveillance capacities of vector-borne diseases and human health in general.

## Figures and Tables

**Table 1:** Main characteristics of breeding sites for primary and secondary species of *Anopheles* mosquitoes transmitting malaria in the Americas, including the Amazon (from [12]).

Mosquito Species	Light Intensity	Salinity	Turbidity	Water Movement	Vegetation	Vector Status	Distribution
*An. darlingi*	low	low	clear water	variable	yes	primary	Entire Amazon region
*An. nuneztovari*	variable	low	clear water	variable	yes	secondary	Brazilian Amazon, Venezuela
*An. marajoara*	variable	low	clear water	variable	yes	secondary	Brazilian Amazon, Venezuela
*An. albitarsis*	high	low	variable	still or stagnant	yes	secondary	Brazilian Amazon, Venezuela
*An. braziliensis*	low	low	clear water	variable	yes	secondary	Brazilian amazon exclusively
*An. oswaldoi*	low	low	clear water	variable	yes	secondary	Brazilian Amazon, Colombia, Peru, Venezuela
*An. trianulatus*	low	low	clear water	variable	yes	secondary	Brazilian Amazon, central and south America

**Table 2:** SAR sensors currently in operation or to be launched in the next few years and their main characteristics. ESA = European Space Agency, DLR = German Aerospace Center, ISA = Italian Space Agency, CSA = Canadian Space Agency, JAXA = Japanese Space Agency, ISRO = Indian Space Research Organization, KARI = Korean Aerospace Research Institute, ANCSA = Argentina National Commission for Space Activities, CNES = French Space Agency, NASA = National American Space Agency, UKSA = United Kingdom Space Agency.

SAR Satellite	Space Agency	In Operation Since	Spatial Resolution (m)	Revisit Period (days)	Scene Size (km^2^)	Band	Polarization	Cost
Sentinel 1A and 1B	ESA	2014 and 2016	5 to 20	5 to 12	20 × 20 to 250 × 200	C	dual	free
TerraSAR-X—TanDEM-X—PAZ	DLR	2007, 2010 and 2014	0.5 to 40	4 to 7	4 × 8 to 270 × 200	X	full	not free
Cosmo-SkyMed	ISA	2007	1 to 100	16	10 × 10 to 200 × 200	X	dual	not free
Radarsat-2	CSA	2007	1 to 100	24	25 × 25 to 500 × 500	C	full	not free
ALOS-2	JAXA	2014	1 to 100	14	25 × 25 to 500 × 500	L	full	not free
Risat-1	ISRO	2012	1 to 50	25	10 × 10 to 225 × 225	C	full	not free
Kompsat-5	KARI	2013	1 to 20	28	5 × 5 to 100 × 100	X	single	not free
SAOCOM-1A and 1B	ANCSA	to be launched in 2018	7 to 100	8 to 16	50 × 50 to 400 × 400	L	full	not free
Radarsat Constellation	CSA	to be launched in 2018	1 to 100	1 to 4	25 × 25 to 500 × 500	C	full	not free
BIOMASS	ESA	to be launched in 2020	50 to 200	17	50 × 50	P	full	not free
SWOT	CNES, NASA, CSA, UKSA	to be launched in 2021	50 to 1000	16	100 km wide-swath	Ka	single	not free
Cosmo-SkyMed SG	ISA	to be launched in 2018	1 to 40	16	10 × 10 to 100 × 3000	X	full	not free
NISAR	NASA, ISRO	to be launched in 2020	3 to 50	12	10 × 10 to 200 × 200	S, L	full	not free

**Table 3:** Recommendations for the use of SAR properties and techniques in order to characterize wetlands typologies.

SAR Properties and Techniques	Free Water	Herbaceous Wetland	Forested Wetland	Herbaceous vs. Forested Wetland	Water Level
**SAR Properties**					
Wavelength band	X	C	L or C + L	C + L	
Polarization	VV	HH	VV or HH	VH or HV or combination	HH, VH or HV
Incidence angle					
**SAR Techniques**					
Backscatter intensity	Thresholding, texture and segmentation	Multi-temporal approach	Multi-temporal and multi-incidence approach	Seasonal approach	
InSAR					Low temporal baselines
Altimetry					Alone or combined with InSAR or backscatter
Polarimetry				HH + HH/HV decomposition Freeman-Dourden and Claude Poitier decompositions	Alpha angle and multi-temporal approach
**Recommended (Free Access Data)**					
	Sentinel 1	Sentinel 1	Sentinel 1, ALOS-PALSAR	Sentinel 1, ALOS-PALSAR	Sentinel 1, ALOS-PALSAR

Note: VV: vertical transmit and vertical receive polarization, HH: horizontal transmit and horizontal receive polarization, HV: horizontal transmit and vertical receive polarization and VH: vertical transmit and horizontal receive polarization.

**Table 4:** A summary of studies using SAR remote-sensing and Altimetry for the characterization of wetland types in the Amazon region.

RADAR Technology	Sensor	Main Results	Product	Reference
Airborne SAR		Classification of vegetation and wetlands	Periodic wetland maps	[74]
C- and L-band SAR	SIR-C	Discrimination between flooded and non-flooded forest in the Amazon	Extent of flooded forest in the Amazon basin	[49]
L-band SAR	JERS-1	Mapping of forest wetlands using L-band SAR	Maps of wetland extent in the central Amazon region	[39]
C- and L-band SAR	ALOS PALSAR + RADARSAT-2	Usefulness of the C-band for the characterization of herbaceous wetlands and improvement of classification accuracy by combining C- and L-bands	Pixel-based classification of wetlands	[58]
SAR interferometry + altimetry	JERS-1 + TOPEX/POSEIDON	Detection of centimeter scale variations of the water surface height in lake Balbina		[67]
L-band SAR	JERS-1	Backscatter values and radar texture for floodplain forest, mangrove forest, flooded forests, dense canopy forest	Regional vegetation and wetland maps	[75]
C-band SAR	RADARSAT-1	Backscatter values and radar texture for classification of various types of mangroves	Mangrove mapping	[76]
L-band SAR	ALOS-PALSAR	Classification of flooded vegetation using the segmentation of a multi-temporal, dual polarization image stack and temporal backscattering signatures	Classification of wetland habitats	[39]
Altimetry	ENVISAT	Time and space variations in water stored in or circulating through rivers, floodplains, wetlands and lakes in the major sub-basins of the Amazon basin	Water storage and circulation in the Amazon basin	[77]
C- and L-band SAR	ALOS PALSAR + RADARSAT-2	Map ecosystems and create a 3-level lake distribution map of the Lower Nhecolàndia subregion in the Brazilian Pantanal	First line spatial resolution classification showing the spatial distribution of terrestrial and aquatic habitats for the entire subregion of Lower Nhecolàndia	[78]
L-band SAR	ALOS-PALSAR	Quad-polarization performs better for forest classification and classification accuracy improves when SAR and optical imagery are combined	Forest classification in the Amazon	[79]
L-band SAR	ALOS-PALSAR	Classification if the state of inundation of the entire Amazon using ScanSAR mode	Inundation maps of the Amazon basin	[80]
L-band SAR	ALOS-PALSAR	Classification of wetland areas into five land-cover classes using dual-season backscattering values	Extent of flooded areas in the Amazon basin during low and high water seasons	[81]
